# Assessment of Countermovement Jump: What Should We Report?

**DOI:** 10.3390/life13010190

**Published:** 2023-01-09

**Authors:** Zdravko Anicic, Danica Janicijevic, Olivera M. Knezevic, Amador Garcia-Ramos, Milos R. Petrovic, Dimitrije Cabarkapa, Dragan M. Mirkov

**Affiliations:** 1 Faculty of Sport and Physical Education, The Research Centre, University of Belgrade, 11000 Belgrade, Serbia; 2 Department of Physical Education and Sport, Faculty of Sport Sciences, University of Granada, 18011 Granada, Spain; 3 Department of Sports Sciences and Physical Conditioning, Faculty of Education, Universidad Católica de la Santísima Concepción, Concepción 4030000, Chile; 4 Jayhawk Athletic Performance Laboratory—Wu Tsai Human Performance Alliance, University of Kansas, Lawrence, KS 66045, USA

**Keywords:** force platform, kinematic, kinetic, testing, vertical jump

## Abstract

The purpose of the present study was (i) to explore the reliability of the most commonly used countermovement jump (CMJ) metrics, and (ii) to reduce a large pool of metrics with acceptable levels of reliability via principal component analysis to the significant factors capable of providing distinctive aspects of CMJ performance. Seventy-nine physically active participants (thirty-seven females and forty-two males) performed three maximal CMJs while standing on a force platform. Each participant visited the laboratory on two occasions, separated by 24–48 h. The most reliable variables were performance variables (CV = 4.2–11.1%), followed by kinetic variables (CV = 1.6–93.4%), and finally kinematic variables (CV = 1.9–37.4%). From the 45 CMJ computed metrics, only 24 demonstrated acceptable levels of reliability (CV ≤ 10%). These variables were included in the principal component analysis and loaded a total of four factors, explaining 91% of the CMJ variance: performance component (variables responsible for overall jump performance), eccentric component (variables related to the breaking phase), concentric component (variables related to the upward phase), and jump strategy component (variables influencing the jumping style). Overall, the findings revealed important implications for sports scientists and practitioners regarding the CMJ-derived metrics that should be considered to gain a comprehensive insight into the biomechanical parameters related to CMJ performance.

## 1. Introduction

The countermovement jump (CMJ) is one of the most implemented testing modalities for the assessment of lower body mechanical capacities. It has been primarily used for monitoring sports performance [1], inter-limb asymmetries [2], neuromuscular fatigue [3], and the effectiveness of different training programs [4]. The main reasons for the widespread use of the CMJ may be attributed to the simplicity of the testing protocols and the higher ecological validity when compared to other traditionally used testing methods (e.g., isokinetic or isometric tests). Moreover, the global market offers a variety of devices, from more rudimentary (e.g., meter-scale-based devices) to highly sophisticated ones (e.g., high-frequency motion capture systems), which facilitate the computation of different CMJ metrics. However, despite the exponential growth of performance monitoring technologies, force platforms are still considered the gold standard testing modality for the assessment of CMJ performance, since they allow sports scientists and practitioners to obtain a plethora of biomechanical variables with high levels of reliability [5,6].

The CMJ variables computed using force platforms can be roughly categorized into: (i) performance, (ii) kinetic, and (iii) kinematic. Undoubtedly, performance variables are the most frequently considered in the scientific literature and practical setting [7]. One variable that stands out from all performance metrics is jump height, due to its direct association with the performance on a variety of sport-specific tasks and physical preparedness of the athletes [3,8]. Moreover, some complex metrics, such as leg stiffness (i.e., ratio of vertical ground reaction force to minimal centre of mass displacement) and modified reactive strength index (RSI modified; i.e., quotient of jump height and push-off duration), are gaining massive popularity in terms of being considered as indirect measures of elastic/reactive properties of lower body muscles [9,10,11]. Kinetic variables (e.g., mean force, peak force, impulse) have been frequently computed by sports scientists and practitioners due to their ability to provide in-depth insight into the mechanics of the CMJ execution [12]. On the other hand, kinematic variables (e.g., jump duration, time to peak force, propulsive phase duration) have been analyzed to quantitatively portray the jump strategy used to achieve a given jump performance. However, they seem to receive less attention in the scientific literature when compared to the performance and kinetic variables [13].

The practical utility of CMJ-derived variables is largely contingent on their reliability. In addition to the natural variability of the human system (i.e., biological variability), the reliability of the CMJ-derived variables is greatly affected by the complexity of the computational methods. In other words, variables that require a greater number of computational steps (i.e., kinematic variables) tend to have lower reliability, while the variables directly calculated from the force–time curve usually display higher levels of reliability (i.e., performance and kinetic variables) [14]. For instance, good-to-excellent reliability was reported for the jump height and RSI modified (performance variables) [15,16,17,18], mean and peak force, power and breaking impulse (kinetic variables) [14,16,19], and some kinematic variables (i.e., peak propulsive velocity and jump depth) [16,19,20]. Other kinematic variables, such as the duration of the unloading, breaking, and landing phases, revealed poor levels of reliability [5,14,16,21]. Moreover, it has been generally argued that variables derived from the downward portion of the force–time curve have lower reliability than the variables derived from the upward portion [14,19], while there is no consensus regarding the reliability of the duration of the propulsive phase and peak breaking velocity [14,16,19,21]. 

Several studies evaluated the possibility and usefulness of reducing the large pool of CMJ-derived variables to a more pragmatical number of essential variables for describing and understanding the overall CMJ performance [22,23,24,25]. This has been usually performed by applying factor analysis to all computed CMJ metrics and defining the number of factors, as well as their structure (i.e., corresponding variables). Interestingly, different studies reported between two and four main factors with a certain difference in the structure of the variables that are loading different factors. For example, among the studies that identified two main factors, Laffaye et al. [22,23] identified the rate of force development (RFD) as a force factor, while Kipp et al. [24] identified RFD as a velocity factor. Some authors reported that overall CMJ variance should be explained by more than two factors [26]. Likewise, Merrigan et al. [27] identified three main factors (having military personnel as the group of subjects), where the first factor was loaded by the variables defining potential for reaching jump height (e.g., mean propulsive force, RSI modified), second by the variables responsible for the strategy to reach a high jump (e.g., velocity, jump depth), and third by the variables defining overall jump outcome (e.g., jump height). Moreover, the same group of authors identified four relevant factors when examining elite collegiate athletes as a cohort of targeted participants [25]. In addition to the study sample, the possible explanation for discrepancies in the number of factors and their structure may be attributed to the different number of CMJ-derived variables considered for the factor analysis in the aforementioned studies (usually without considering the variables related to the landing phase of the jump). 

To solve this problem, 45 force–time-derived variables were identified as potentially important CMJ metrics, and the aims of this study were (i) to explore their within- and between-day reliability, and (ii) to reduce a large pool of identified CMJ-derived variables with acceptable levels of reliability to the significant factors using principal component analysis. From the reliability standpoint, it has been hypothesized that the CMJ variables will be ranked from most to least reliable as follows: performance variables > kinetic variables > kinematic variables. However, the hypothesis regarding the minimum number of factors that can explain the overall CMJ performance could not be set due to the inconsistent findings previously reported in the scientific literature. The results of the present study are expected to reveal the list of highly reliable metrics that should be used to thoroughly explore the lower body neuromuscular capacities through the CMJ. 

## 2. Materials and Methods

### 2.1. Participants

Seventy-nine physically active participants (37 women (body mass = 65.7 ± 10.5 kg, height = 1.72 ± 0.01 m, age = 22.2 ± 3.6 years) and 42 men (body mass = 79.3 ± 9.5 kg, height = 1.85 ± 0.07 m, age = 22.4 ± 4.1 years)) volunteered to participate in the present study. All participants had at least one year of lower-body resistance training experience and most of them were actively involved in resistance training at the time of the testing. Moreover, they were free of musculoskeletal injuries and/or pain that could negatively impact CMJ performance, and none of them were taking any supplementation at the time of the study. Participants were familiarized with the research protocol in both written and verbal manner. The study was conducted according to the guidelines of the Declaration of Helsinki and approved by the Institutional Review Board of the University of Belgrade, Faculty of sport and physical education (protocol number: 02-273/21-1, date: 16 March 2021).

### 2.2. Study Design

The present study aimed to explore the reliability of the most frequently used CMJ variables and to provide a list of reliable metrics that are necessary to be considered when exploring lower body neuromuscular capacities through CMJ. For this purpose, participants completed two identical testing sessions, separated 24–48 h apart. During each experiment, participants performed three maximal CMJs without arm swing (having their hands fixed on the hips). The rest interval between each CMJ was 60 s [18]. The sessions were performed under identical laboratory conditions for all subjects.

### 2.3. Testing Protocol

All testing procedures were performed in the university research laboratory and at the same time of the day for each subject (±30 min). The participants were required to avoid any intense physical activity 24 h prior to each testing session to ensure their full readiness for the testing. Upon arrival at the laboratory, participants performed a standardized warm-up procedure consisting of 5 min of stationary cycling at a self-selected pace and a set of dynamic exercises (e.g., calf raises, hip hinges, lunges, squats, and hopping), followed by five submaximal CMJs with a 30 s inter-jump rest interval. Two minutes after the completion of the warm-up protocol, participants stepped on a previously calibrated force plate wearing their habitual training shoes during both sessions. They were instructed to stand steadily for 5 s, jump as high as possible after the “go” signal given by the research assistant, and land at approximately the same spot on the force plate. Each participant completed three maximal CMJs using a self-selected countermovement depth. The rest interval between consecutive CMJ was 60 s [28,29]. The same protocol was repeated during the second session. Verbal encouragement was provided systematically for every CMJ. The highest jump from the first and second sessions were recorded and incorporated into the between-day reliability analysis. 

### 2.4. Data Processing

Vertical ground reaction force (vGRF) data were recorded using fixed bilateral three-dimensional force platforms (AMTI BP600400, Watertown, MA, USA) at a sampling frequency of 1000 Hz. The vGRF data obtained from both platforms was summed and processed using custom-made software (MATLAB and Statistics Toolbox Release 2015a, The MathWorks, Inc., Natick, MA, USA). Body weight was established during the 2 s motionless period prior to the beginning of the downward phase of the CMJ motion. The initiation of the downward phase was identified as the moment when the force–time curve trace dropped 10 N below body weight, the take-off when the vGRF was below 5 N, and the landing as the point where vGRF exceeded the 5 N threshold. The centre of mass (COM) acceleration was calculated as the net vGRF (absolute vGRF—body weight) divided by the participant’s body mass. In addition, COM velocity was calculated as a numeric integration of acceleration data with respect to time and COM displacement as a double integral of COM acceleration. All force-derived variables were scaled to the subject’s body mass. Impulse-momentum and flight time methods were used to calculate jump height [17]. RSI was calculated by dividing jump height by the time duration between initiation of the downward phase and the take-off [20]. Likewise, leg stiffness was calculated as the ratio between peak breaking force and COM displacement during the breaking phase. 

**Figure 1:**
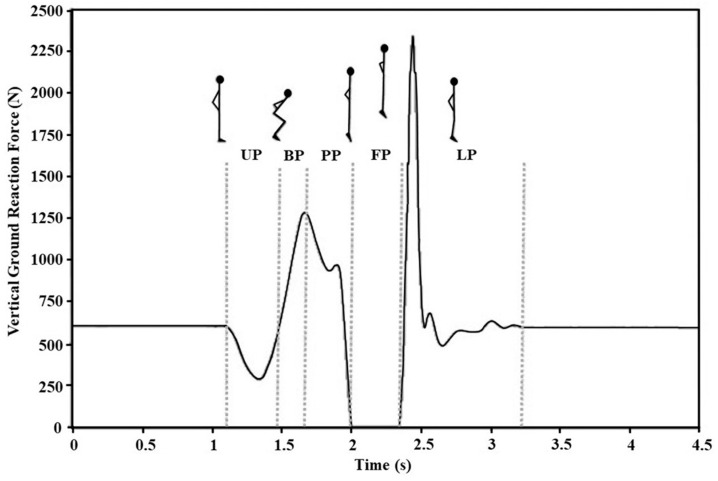
Typical vertical ground reaction force–time signal of the countermovement jump obtained from the force (y axis represents vertical ground reaction force [N], while x axis represents time [ms]). Vertical dotted lines divide typical phases of the countermovement jump (UP, unloading phase; BP, breaking phase; PP, propulsive phase; FP, flight phase, LP, landing phase).

Additionally, CMJ force–time curve was calculated for the following five phases (Figure 1): (i) Unloading phase—a portion of the force–time curve portion between jump initiation and minimum vGRF; (ii) Breaking phase—a part of the force–time curve between reaching the minimum vGRF and reaching the lowest downward displacement of the COM; (iii) Propulsive phase—a portion of the force–time curve from reaching the lowest COM position until the take-off; (iv) Flight phase—the time in the air between the take-off and the first contact during landing; and (iv) Landing phase—the portion of the force–time curve beginning with the initiation of the landing and the moment when the COM velocity is 0 m·s^−1^. The list of all CMJ-derived dependent variables (45 total), categorized into performance, kinetic, and kinematic variables, is presented in Table 1.

### 2.5. Statistical Analysis

Intraclass correlation coefficient (ICC; model 3.1) and coefficient of variation (CV%) were used for assessing within- and between-day reliability for the 45 dependent variables computed in this study. Acceptable reliability was determined as an ICC ≥ 0.70 and CV ≤ 10% [30]. Paired-sample t-tests were used to detect differences between sessions and uncover the possible learning effect. The magnitude of the change was determined using Cohen’s *d* effect size (ES) and interpreted using the criterion proposed by Hopkins [31]: trivial <0.20; small = 0.20–0.59; moderate = 0.60–1.19; large = 1.20–2.00; and extremely large >2.00. Repeated measures ANOVA was used for comparing the magnitude of the three jumps performed within each day. Correlations between the metrics and partial correlations relative to the full correlations of data were assessed using Bartlett’s test of sphericity and the Kaiser–Mayer–Olkin (KMO) measurement. Then, principal component analysis (PCA) with Varimax rotation was used to extract principal components, based on Eigenvalue (i.e., the total amount of variance that can be explained by a given principal component) >1 [32]. All statistical analyses were performed using the software package SPSS (Version 25.0; IRB Corp., Armonk, NY, USA) and Microsoft Excel (Microsoft Corp., Redmond, WA, USA).

## 3. Results

**Figure 2:**
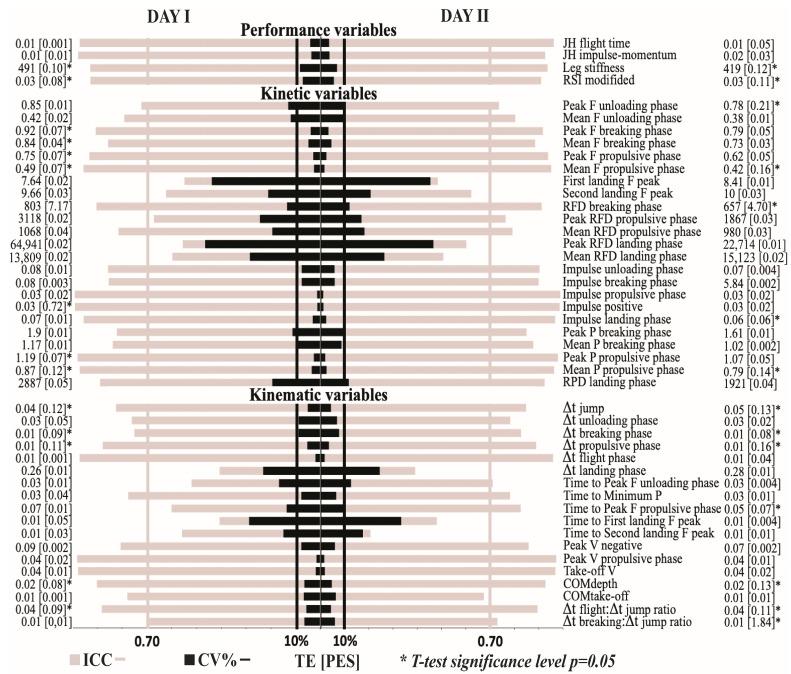
Within-day reliability of the different variables derived from the countermovement jump test. Gray bars indicate the magnitude of the interclass correlation coefficient (ICC), while black bars indicate the coefficient of variation (CV%) for the first (left side of the figure) and the second day (right side of the figure). Force, impulse, and power variables are scaled to body mass. Vertical black lines delimit the area of acceptable reliability based on CV = 10%, while gray delimit acceptable reliability based on ICC = 0.70; PES, partial eta square, * denotes ANOVA significance level (*p* ≤ 0.05). JH, jump height; RSI modified, reactive strength index—modified; F, force; RFD, rate of force development; P, power; Δt, duration of the certain phase; V, velocity; COM, center of mass.

**Figure 3:**
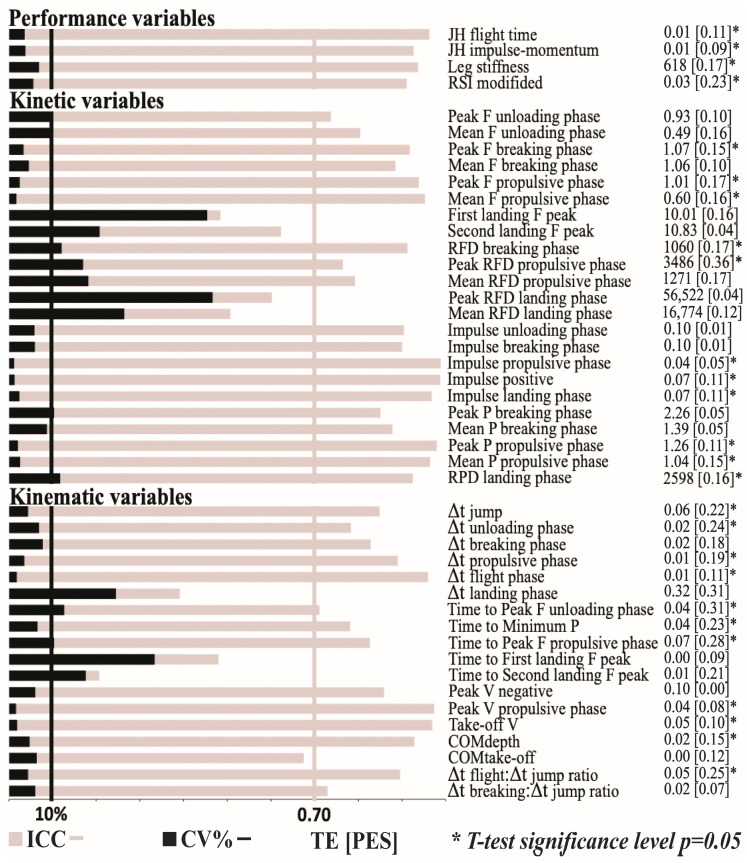
Between-day reliability of the countermovement jump. Gray bars indicate the magnitude of the interclass correlation coefficient (ICC), while black bars indicate coefficient of variation (CV). Vertical black lines delimit the area of acceptable reliability based on CV = 10%, while gray delimit acceptable reliability based on ICC = 0.70; TE, typical error of measurement; ES, effect size, * denotes that t-test was significant at *p* ≤ 0.05. JH, jump height; RSI modified, reactive strength index—modified; F, force; RFD, rate of force development; P, power; Δt, duration of the certain phase; V, velocity; COM, center of mass.

Within- and between-day reliability of the performance, kinetic, and kinematic variables are presented in Figure 2 and Figure 3, respectively. All performance variables presented acceptable within- and between-day reliability (CV ≤ 8.57%) with the only exception being leg stiffness, which presented a lower between-day reliability (CV = 11.1%). Regarding kinetic variables, the impulse-related variables always presented an acceptable reliability (CV ≤ 8.3%); force- and power-related variables were generally more reliable for the propulsive phase (CV ≤ 4.35%) than for the unloading, breaking, and landing phases (CV ≤ 44.3%); and RFD-related variables never reached an acceptable level of reliability (CV ≥ 22.2%). Finally, only 8 out of 18 kinematic variables reached acceptable levels of reliability: all velocity variables (CV ≤ 8.5%), depth of the countermovement and height of the COM at take-off (CV ≤ 8.2%), two variables that describe the duration of the propulsive (CV = 1.9%) and flight phases (CV = 2.2%), and the variable that describes the ratio between flight time and jump time (CV = 8.3%). 

The differences in the magnitude of the variables between sessions 1 and 2 were significant for all performance variables, for 11 out of 23 kinetic variables, and for 11 out of 18 kinematic variables. However, of all the 26 variables that differed between sessions 1 and 2, only 6 presented a higher magnitude during the second testing session (time to reach maximal force during unloading and propulsive phases, time to reach minimal power, depth of the COM, and the duration of the unloading and propulsive phases). Nevertheless, the ES always ranged from trivial to small (ES = 0.05–0.36; Figure 3). 

**Figure 4:**
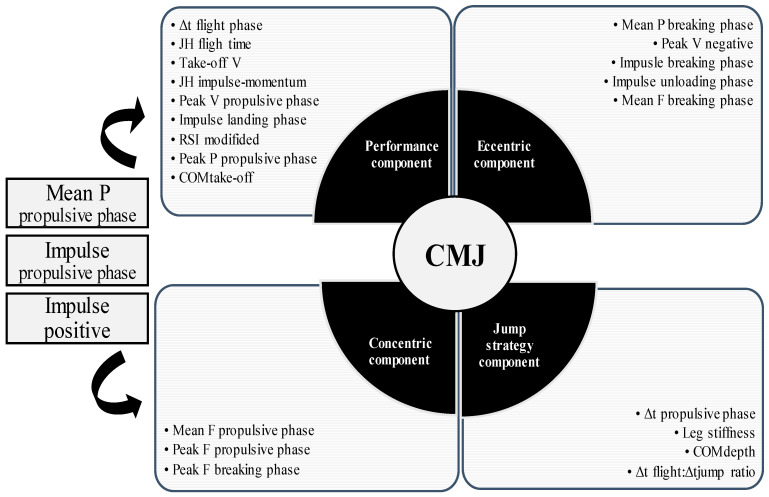
Principal component analysis output. CMJ, countermovement jump; P, power; Δt, duration of the certain phase; JH, jump height; V, velocity; RSI, reactive strength index; COM, center of mass; F, force.

The KMO measure of sampling (0.799) and Bartlett’s sphericity test (χ^2^ = 6378; *p* < 0.001) suggested sample adequacy and sufficient correlations between variables. Based on Kaiser’s criterion factor analysis, four principal components were extracted which explained 91.8% of overall CMJ variance (Table 2; Figure 4). Generally, the first factor was loaded by the variables related to the propulsive and flight phases (performance component) and explained 59% of the variance. The second factor was loaded by the variables related to the breaking phase (eccentric component) and explained 16% of the variance. The third factor was loaded with the variables related to the propulsive and breaking phases (concentric component) and explained 11% of the variance. The fourth factor was loaded by the kinematic variables, which are generally related to the jump strategy (jump strategy component) and explained 6% of the variance. Interestingly, three variables (mean propulsive power, propulsive impulse, and total positive impulse) overlapped both the first and third factors based on loading values.

## 4. Discussion

The purpose of the present study was twofold: (i) to assess the reliability of a large number of performance, kinetic, and kinematic CMJ-derived variables and (ii) to provide a reduced list of reliable metrics that should be reported to provide information regarding the distinctive aspects of CMJ performance. The main findings revealed that only 24 out of 45 CMJ-derived variables demonstrated acceptable within- and between-day reliability and that the most reliable metrics were the performance variables (3 out of 4), followed by the kinetic variables (12 out of 23), and finally the kinematic variables (8 out of 18). Four main components were extracted as a result of principal component analysis applied to the 24 reliable CMJ-derived variables and were conveniently addressed as: performance component, eccentric component, concentric component, and jump strategy component, explaining 56%, 16%, 11%, and 6% of the common variance, respectively. These findings present important implications for sports scientists and practitioners regarding the CMJ-derived metrics that should be considered to gain a comprehensive insight into the distinctive aspects of the CMJ performance.

All performance variables were obtained with an acceptable within- and between-day reliability with the only exception being leg stiffness, which presented a between-day reliability lower than the minimal threshold for acceptable reliability (CV > 10%). These results are in line with the findings of previous research reports focused on exploring the reliability of the CMJ-derived performance variables [16,19,21]. For instance, exceptional reliability was obtained in the present investigation for jump height, regardless of the computational method (impulse-momentum or flight time approaches), which is in agreement with several recent studies [16,19,21] that reported CV < 5% for jump height. Moreover, the RSI modified, considered an important indicator of neuromuscular function, showed an acceptable reliability with a CV of 8.6%; despite this, we did not fix the countermovement depth. The acceptable reliability of the RSI modified is in line with the findings of previous studies [18,20,33] (CV ≤ 10%). The only performance variable that presented a questionable reliability was the leg stiffness (CV = 11%), a variable that is considered as a quantitative measure of the elastic properties of lower body muscles [9]. Although several studies reported similar findings [17,19,34], Heishman et al. [21] argued that leg stiffness cannot be considered a reliable CMJ-derived variable because they obtained CV values that exceeded 20%. A possible explanation for the discrepancies between studies regarding the reliability of leg stiffness could be the different CMJ strategies implemented by the participants [33,35], as well as the gender and participant’s sports background (e.g., familiarity with the CMJ). 

Regarding kinetic variables, impulse-related variables were the most reliable (all CV ≤ 8.3%), followed by force and power variables (50% of variables with acceptable reliability in each group), while RFD-related variables never reached an acceptable level of reliability. Impulse-related variables were also considered reliable metrics in previous studies [7,17,36], which is an important finding, since they are responsible for the jump height and jump mechanics in general [29,37]. On the other hand, force-related variables, although obtained from the directly recorded force–time curve, were not always obtained with acceptable reliability. For instance, variables related to the breaking and propulsive phase demonstrated acceptable reliability (CV ≤ 6.1%), while variables related to the unloading and landing phase did not meet the criteria for acceptable reliability. These results are in line with previous evidence [14,19,21]. Since the computational method was rather direct, the low reliability of the kinetic variables collected during the unloading and landing phases might be explained by the high variability produced when participants are allowed to self-select the countermovement depth. Furthermore, power-related variables collected during the breaking phase showed lower reliability (CV ≥ 10.9%) compared to the variables collected during the propulsive phase (CV ≤ 4.1%), which is contrary to the findings of Merrigan et al. [19] who reported acceptable levels of reliability for the CMJ-derived power variables collected during the breaking phase of CMJ motion. Finally, although RFD-related variables are considered important metrics for athletes who have limited time to exert force and are frequently considered for monitoring the neuromuscular component of the human movement [14,21,29,38], none of the RFD-related variables reached acceptable reliability in the present study, questioning their use when assessing lower-body neuromuscular performance through the execution of the CMJ.

Confirming our first hypothesis, only 8 out of 18 kinematic variables presented acceptable reliability. Specifically, all velocity- and COM-related variables were reliable, as well as the duration of the propulsive phase, flight phase, and the ratio between flight time and overall jump time. In line with our study, velocity-related variables were shown to be consistently reliable [19,21,39], as well as the COM-related variables (depth of the countermovement and height of the COM at take-off) [14,19,39]. However, the results of previous studies are somewhat contradictory when it comes to the reliability of the duration of the different CMJ phases. Contrary to the findings of the present study, Warr et al. [14] and Heishman et al. [21] reported low reliability for the propulsive phase duration, while the breaking phase duration was sometimes found to be reliable [14,27] but other times was not a reliable metric [21]. The duration of the flight phase seems to be universally accepted as the most reliable metric when it comes to the duration of different CMJ phases [21] because the parameters that are influencing jump height are consistent within the same participants (i.e., similar velocities at take-off and correct jumping technique). On the other hand, the duration of the landing phase and overall duration of the jump were not studied extensively. The variables related to the time necessary to reach certain mechanical peaks on the CMJ curve were generally considered non-reliable, as shown in our study [14].

In summary, a total of 24 (out of 45) CMJ-derived variables demonstrated acceptable reliability and, therefore, were included in the principal component analysis. Considering the number of the included variables and extracted factors, our study is similar to the study of Merrigan et al. [25] who included 19 variables in the principal component analysis and extracted the same number of factors (i.e., four factors). However, the variables that load the four factors in the study of Merrigan et al. [25] differ from our study. Specifically, our 24 variables were grouped into a performance component (loaded by the variables related to the propulsive and flight phases), eccentric component (loaded by the variables related to the breaking phase), concentric component (loaded by the variables related to the concentric phase), and jumps strategy component (loaded by the variables specific for selecting a different jump strategy). Although Merrigan et al. [25] also extracted factors related to the concentric phase and another factor related to overall jump performance, two other factors were loaded with the variables related to the breaking phase. Another important discrepancy between our and the findings of Merrigan et al. [25] is that they did not extract any factor related to the jump strategy. The reason why our findings cannot be directly compared to other studies is due to a lower number of CMJ-related variables (i.e., 11 or 15) included in the analysis procedures [26], as well as due to the CMJ-related variables that were excluded from our principal component analysis because of their lack of reliability (e.g., breaking phase duration, breaking RFD, power during breaking phase). The explanation for why breaking phase-related variables showed low reliability is possibly because they are diverse strategies adopted for performing the downward phase of the CMJ (i.e., different jump depth and lowering velocities).

Several limitations of the present study should be acknowledged. First, the sample consisted of physically active young participants engaged in recreational physical activities, and it is unknown whether the results of the present study could be generalized to other populations (e.g., athletes or sedentary individuals). Second, it is important to acknowledge the low number of subjects included in the study per variable [40], although the total number of participants in our study was larger/similar when compared to other research reports (*n* = 79 vs. *n* = 16–82) [25,26,27]. Third, it should be noted that the principal component analysis included two variables whose reliability was on the border of the acceptability criteria (breaking mean power [CV = 10.85%] and leg stiffness [CV = 11.01%]). 

## 5. Conclusions

From the large pool of 45 CMJ-derived variables computed in the present study, only 24 demonstrated acceptable within- and between-day levels of reliability. The CMJ-derived variables ranked in the order of highest to lowest reliability magnitude were as follows: performance variables (e.g., jump height, modified reactive strength index), kinetic variables (impulse-related variables were the most reliable), kinematic variables (only 8 out of 18 kinematic variables revealed acceptable reliability). When included into the principal component analysis, these 24 variables loaded four factors, explaining 91% of the variance and were conveniently addressed as performance component (loaded by the variables responsible for overall jump performance), eccentric component (loaded by the variables related to the breaking phase of the CMJ), concentric component (loaded by the variables related to the concentric phase of the CMJ) and jump strategy component (loaded by the variables that are importantly influencing the jumping style, such as the depth of the countermovement). Overall, the findings of the present study reveal important implications for sports scientists and practitioners regarding the CMJ-derived variables that should be considered to gain a comprehensive insight into the mechanics pertaining to CMJ performance.

## Figures and Tables

**Table 1:** List of all the 45 countermovement-jump-derived variables computed in this study along with their units of measurement.

Variable Type	Variable	Unit
Performance variables	Jump height (flight time method)	cm
	Jump height (impulse momentum method)	cm
	Leg stiffness	AU
	Reactive strength index—modified	AU
Kinetic variables	Unloading peak force	N
	Unloading mean force	N
	Breaking peak force	N
	Breaking mean force	N
	Propulsive peak force	N
	Propulsive mean force	N
	Landing first force peak	N
	Landing second force peak	N
	Breaking rate of force development	N·s^−1^
	Propulsive peak rate of force development	N·s^−1^
	Propulsive mean rate of force development	N·s^−1^
	Landing peak rate of force development	N·s^−1^
	Landing mean rate of force development	N·s^−1^
	Unloading impulse	N·s
	Breaking impulse	N·s
	Propulsive impulse	N·s
	Positive impulse	N·s
	Landing impulse	N·s
	Breaking peak power	W
	Breaking mean power	W
	Propulsive peak power	W
	Propulsive mean power	W
	Landing rate of power development	W·s^−1^
Kinematic variables	Jump duration	s
	Unloading phase duration	s
	Breaking phase duration	s
	Propulsive phase duration	s
	Flight phase duration	s
	Landing phase duration	s
	Time to unloading peak force	s
	Time to minimum power	s
	Time to propulsive peak force	s
	Time to first landing force peak	s
	Time to second landing force peak	s
	Peak negative velocity	m·s^−1^
	Propulsive peak velocity	m·s^−1^
	Take-off velocity	m·s^−1^
	Countermovement center of mass depth	cm
	Center of mass at take off	cm
	Flight time:Jump time ratio	AU
	Breaking time:Jump time ratio	AU

Note: AU—arbitrary units.

**Table 2:** Components extracted by principal component analysis, including loading and communalities values of the contributing countermovement jump variables.

Variables	Performance Component	Eccentric Component	Concentric Component	Jump Strategy Component	Communalities
Δt flight phase (s)	**0.936**				*0.968*
JH flight time (cm)	**0.934**				*0.967*
Take-off V (m·s^−1^)	**0.924**				*0.980*
JH impulse-momentum (cm)	**0.924**				*0.980*
Peak V propulsive phase (m·s^−1^)	**0.919**				*0.981*
Impulse landing phase (N·s)	**0.763**				*0.903*
RSI modified (AU)	**0.755**				*0.952*
Peak P propulsive phase (W)	**0.745**				*0.954*
Mean P propulsive phase (W)	0.693		0.625		*0.984*
Impulse propulsive phase (N·s)	0.673		0.671		*0.985*
Impulse positive (N·s)	0.673		0.672		*0.986*
COM take-off (m·s^−1^)	**0.554**				*0.325*
Mean P breaking phase (W)		**0.929**			*0.992*
Peak V negative (m·s^−1^)		**−0.921**			*0.985*
Impulse breaking phase (N·s)		**0.908**			*0.985*
Impulse unloading phase (N·s)		**−0.900**			*0.980*
Mean F breaking phase (N)		**0.805**			*0.946*
Mean F propulsive phase (N)			**0.826**		*0.982*
Peak F propulsive phase (N)			**0.815**		*0.920*
Peak F breaking phase (N)			**0.693**		*0.906*
Δt propulsive phase (s)				**−0.919**	*0.926*
Leg stifness (AU)				**0.812**	*0.696*
COM depth (cm)				**0.735**	*0.880*
ΔtFP:Δtjump ratio (AU)				**0.672**	*0.871*
**Eigenvalues**	14.1	3.9	2.7	1.3	
**% of Variance**	59	16	11	6	

Bold numbers indicate that the variable dominantly belongs to only one component. Italic numbers present communalities (i.e., the proportion of each variable’s variance that can be explained by the factors). Eigenvalues present the total amount of variance that can be explained by a given principal component. Δt, duration of the certain phase; JH, jump height; V, velocity; RSI modified, reactive strength index—modified; P, power; COM, center of mass; F, force.

## Data Availability

Data available on request due to privacy restrictions.
